# Mechanism of suppression of sustained neuronal spiking under high-frequency stimulation

**DOI:** 10.1007/s00422-013-0567-1

**Published:** 2013-10-22

**Authors:** Kestutis Pyragas, Viktor Novičenko, Peter Alexander Tass

**Affiliations:** 1 Center for Physical Sciences and Technology, 01108 Vilnius, Lithuania; 2 Institute of Neuroscience and Medicine-Neuromodulation (INM-7), Research Center Jülich, 52425 Jülich, Germany; 3 Department of Neuromodulation, Cologne Medical School, 50924 Cologne, Germany; 4 Clinic for Stereotatic and Functional Neurosurgery, University of Cologne, 50924 Cologne, Germany

**Keywords:** High-frequency deep brain stimulation, Method of averaging, Parkinson’s disease, Hodgkin–Huxley model, Subthalamic nucleus model neuron

## Abstract

Using Hodgkin–Huxley and isolated subthalamic nucleus (STN) model neurons as examples, we show that electrical high-frequency stimulation (HFS) suppresses sustained neuronal spiking. The mechanism of suppression is explained on the basis of averaged equations derived from the original neuron equations in the limit of high frequencies. We show that for frequencies considerably greater than the reciprocal of the neuron’s characteristic time scale, the result of action of HFS is defined by the ratio between the amplitude and the frequency of the stimulating signal. The effect of suppression emerges due to a stabilization of the neuron’s resting state or due to a stabilization of a low-amplitude subthreshold oscillation of its membrane potential. Intriguingly, although we neglect synaptic dynamics, neural circuity as well as contribution of glial cells, the results obtained with the isolated high-frequency stimulated STN model neuron resemble the clinically observed relations between stimulation amplitude and stimulation frequency required to suppress Parkinsonian tremor.

## Introduction

In the past two decades, great progress has been achieved in the application of high-frequency stimulation (HFS) to biological systems. Deep brain stimulation (DBS) at high frequencies (HF) is the standard therapy for medically refractory Parkinson’s disease (PD) and essential tremor (Benabid et al. 1991; Limousin et al. 1995, 1998; Lozano et al. 2002; Rodriguez-Oroz et al. 2000). Nevertheless, its mechanism of action is still unclear (Benabid et al. 2005; McIntyre et al. 2004a; Vitek 2002). Multiple possible mechanisms may contribute to the therapeutic effects of DBS [for review see Benabid et al. (2002), McIntyre et al. (2004a), Vitek (2002)], e.g., (1) Neuronal activity is blocked because stimulation changes the activation of voltage-gated currents in the vicinity of the stimulating electrode (depolarization blockade) (Beurrier et al. 2001). (2) Neuronal activity near the stimulating electrode is indirectly inhibited via an excitation of axon terminals that are connected with neurons by inhibitory synapses (synaptic inhibition) (Dostrovsky et al. 2000). (3) Stimulation causes a transmitter depletion, which, in turn, leads to a synaptic transmission failure of the efferent output of stimulated neurons (synaptic depression) (Urbano et al. 2002). (4) Stimulation changes the pathological network activity (Montgomery and Baker 2000). (5) HFS forces neurons to fire in a regular manner, so that neurons are prevented from information processing (neuronal jamming) (Benabid 2003; Benabid et al. 2005). Related to this hypothesis, in computational models, it was shown that HFS may induce a high-frequency neuronal activity with zero variance (informational lesion) (Grill et al. 2004). (6) A release of adenosine inhibits neurons (Bekar et al. 2008). (7) Stimulation induces long-term plastic changes (Hauptmann and Tass 2007).

In fact, several of the mechanisms likely contribute to the therapeutic DBS effects (Benabid et al. 2002; Vitek 2002). While some of these mechanisms refer to effects in the vicinity of the stimulating electrode, other effects are mediated by projection neurons (McIntyre et al. 2004a, b): For instance, both depolarization blockade and synaptic inhibition are possible mechanisms underlying the suppression of the somatic firing throughout the target nucleus. In contrast, independently of the suppression of neuronal activity in the vicinity of the stimulating electrode DBS may cause a high-frequency axonal output via projection neurons (McIntyre et al. 2004b).

Our study is devoted to the dynamical mechanism underlying the suppression of neuronal activity on an elementary membrane level. For this, we neglect synaptic and network mechanisms and exclusively study the effects of DBS on a single-compartment conductance-based biophysical subthalamic nucleus (STN) neuron model (Terman et al. 2002). Clinically, it is known that the effects of DBS crucially depend on the relationship between the stimulation frequency and the frequency of the pathological oscillatory activity. For instance, DBS at HF (greater than 100 Hz) effectively suppresses essential tremor and Parkinsonian tremor, whereas low-frequency DBS (lower than 50 Hz) does not lead to a tremor suppression and may even boost the tremor when delivered at frequencies around 5–10 Hz (Benabid 2003; Benabid et al. 1987, 1991). Accordingly, we particularly focus on HFS delivered at sufficiently HF compared to the spontaneous neuronal firing rate (i.e., the neuron’s firing rate in the absence of stimulation). To this end, for the STN model neuron under consideration, we consider stimulation frequencies $>$100 Hz. We demonstrate that a charge-balanced HFS may suppress self-sustained neuronal spiking and explain this effect in terms of a stabilization of the neuron’s resting state. To gain an intuitive understanding of this effect, we refer to a mechanical analogy. It is well known that the behavior of mechanical systems may drastically change under the action of vibration. Vibration may cause various bifurcations such as a creation or destruction of equilibrium points as well as changes in the stability properties of existing equilibrium points (Blekhman et al. 2003). A classical example is the stabilization of the upside-down position of a rigid pendulum by vibrating its pivot up and down at a suitably high frequency. A theoretical approach to solve this problem has been proposed by Kapitsa (1951). We here show that the stabilization of a neuron’s resting state under HFS is analogous to this effect and admits the same mathematical treatment as in vibrational mechanics. In analogy with vibrational mechanics, we separate the neuron dynamics into slow and fast components and show that the resting state becomes stable. The level of description chosen in our study is given by a single-compartment conductance-based biophysical neuron model. Obviously, our approach employs a sort of elementary and simplified modeling level, since we neglect, e.g., synaptic dynamics and neural circuitry. Nevertheless, our results qualitatively reproduce the clinically observed relations between stimulation amplitude and stimulation frequency necessary to suppress Parkinsonian tremor. The dynamical mechanism of HFS-induced suppression of neuronal firing revealed below is relevant in the context of the depolarization blockade mechanism (Beurrier et al. 2001) as well as neural jamming (Benabid 2003; Benabid et al. 2005).

The paper is organized as follows. In Sect. 2, we derive averaged equations for a general single-compartment neuron model, and in Sect. 3, we demonstrate this approach for the classical Hodgkin–Huxley model (1952). The parameters of the HH model correspond to the giant axon of the squid and are not applicable to the specific neurons in the target areas relevant to DBS. However, the HH model is simpler compared to the STN model (Terman et al. 2002) used below and allows us to illustrate our mathematical approach in a more comprehensible manner. In order to relate our analysis to DBS, in Sect. 4, we consider the effect of HFS on an established STN model neuron (Terman et al. 2002), which represents a modified version of the HH model. The paper finishes with discussion and conclusion in Sect. 5.

## Methods

To clarify the mechanism of action of HFS on the membrane of a single neuron and simplify the mathematical description of a neuron model subject to HF stimulation, we refer to the method of averaging (Sanders et al. 2007), which is widely used in various fields of physics including vibrational mechanics. To adapt this method to neural dynamics, let us consider a general single-compartment Hodgkin–Huxley-type neuron model under HFS:

1a $$\begin{aligned} C_m\dot{v}&= F\left( v,\mathbf{w}\right) +a\varphi (\omega t), \end{aligned}$$

1b $$\begin{aligned} \dot{\mathbf{w}}&= \mathbf{G} \left( v,\mathbf{w}\right) . \end{aligned}$$

Here, $C_m$ is the membrane capacitance, and $v$ is the membrane potential. The function $F$ describes the sum of currents flowing through the ion channels. $a \varphi (\omega t)$ is a HFS current, where $a$ is the amplitude and $\omega$ is the cyclic frequency. We consider a general case when $\varphi (\omega t)$ is any $2 \pi$ periodic dimensionless function $\varphi (\omega t+2\pi )=\varphi (\omega t)$ (not necessarily a harmonic signal) with the amplitude of oscillations equal to one. In order to provide a charge-balanced stimulation, we require $\int _0^T \varphi (\omega t) \hbox {d}t =0$, where $T=2\pi /\omega$ is the period of the HFS. Equation (1b) describes the dynamics of a recovery variable $\mathbf{w}$ that generally is a vector variable, $\mathbf{w} = (w_1, \ldots , w_n)$. The function $\mathbf{G}$ represents the ionic channel dynamics. The dimension *n* of the vector variable $\mathbf w$ as well as the functions $F$ and $\mathbf{G}$ are defined by the specific neuron model.

Our aim is to simplify the nonautonomous system (1) for large frequencies $\omega$, when the period $T=2\pi /\omega$ of HF oscillations is much smaller than the characteristic time scale $T_{0}$ of the neuron in the absence of stimulation. Using the small parameter $\omega ^{-1}2\pi /T_{0} \ll 1$, we seek to eliminate the HF term $a\varphi (\omega t)$ and obtain an autonomous system, the solutions of which approximate the original system. First, we change the variables of system (1):

2a $$\begin{aligned}&\!\!\!\!v(t) = V(t)+A\psi (\omega t), \end{aligned}$$

2b $$\begin{aligned}&\!\!\!\mathbf{w}(t) =\mathbf{W}(t) \end{aligned}$$

with

3 $$\begin{aligned} A=a/C_m\omega \end{aligned}$$

and $\psi (\omega t)=\int ^{\omega t}\varphi (s)\hbox {d}s$. In the latter equation, we choose the integration constant so that $\int _0^T \psi (s)\hbox {d}s=0$. Substituting (2) into (1), we derive the following equations for the new variables $V(t)$ and $\mathbf{W}(t)$:

4a $$\begin{aligned} C_m\dot{V}&= F(V+A\psi (\omega t),\mathbf{W}), \end{aligned}$$

4b $$\begin{aligned} \dot{\mathbf{W}}&= \mathbf{G} (V+A\psi (\omega t),\mathbf{W}). \end{aligned}$$

By rescaling the time variable $t=\omega \tau$ (here $\tau$ is the “fast” time), system (4) can be transformed to the standard form of equations as typically used by the method of averaging (Sanders et al. 2007):

5a $$\begin{aligned} C_m\frac{\hbox {d}V}{\hbox {d}\tau }&= \omega ^{-1}F\left( V+A\psi (\tau ),\mathbf{W}\right) \!, \end{aligned}$$

5b $$\begin{aligned} \frac{\hbox {d}\mathbf{W}}{\hbox {d}\tau }&= \omega ^{-1}\mathbf{G} \left( V+A\psi (\tau ),\mathbf{W}\right) \!. \end{aligned}$$

Due to the small factor $\omega ^{-1}$ in the r.h.s. of Eq. (5), the variables $V$ and $\mathbf{W}$ vary slowly while the periodic functions in the r.h.s. oscillate fast. According to the method of averaging (Sanders et al. 2007), an approximate solution of system (5) can be obtained by averaging the r.h.s. of the system over fast oscillations. Specifically, let us denote the variables of the averaged system as $(\bar{v}, \bar{\mathbf{w}})$. They satisfy the equations:

6a $$\begin{aligned} C_m\frac{\hbox {d}\bar{v}}{\hbox {d}\tau }&= \omega ^{-1} \left\langle F(\bar{v}+A\psi (\tau ),{\bar{\mathbf{w}}}) \right\rangle _{\tau }\!, \end{aligned}$$

6b $$\begin{aligned} \frac{\hbox {d}\bar{\mathbf{w}}}{\hbox {d}\tau }&= \omega ^{-1} \left\langle \mathbf{G} (\bar{v}+A\psi (\tau ),\bar{\mathbf{w}}) \right\rangle _{\tau }\!. \end{aligned}$$

Here, the angle brackets denote the averaging over the period of the fast time $\langle (\cdots ) \rangle _{\tau } = (1/2\pi )\int _0^{2\pi } (\cdots ) \hbox {d}\tau$. The method of averaging states that the averaged system (6) approximates the solutions of the system (5) with the accuracy of $O(\omega ^{-1})$, i.e., $V=\bar{v}+O(\omega ^{-1})$ and $\mathbf{W}=\bar{\mathbf{w}}+O(\omega ^{-1})$. After coming back to the original time scale, the averaged system (6) takes the form (where the dot denotes differentiation with respect to the original time *t*):

7a $$\begin{aligned} C_m \dot{\bar{v}}(t)&= \left\langle F(\bar{v}(t)+A\psi (\tau ),{\bar{\mathbf{w}}}(t)) \right\rangle _{\tau }, \end{aligned}$$

7b $$\begin{aligned} {\dot{{\bar{\mathbf{w}}}}}(t)&= \left\langle \mathbf{G} (\bar{v}(t)+A\psi (\tau ),\bar{\mathbf{w}}(t)) \right\rangle _{\tau }. \end{aligned}$$

Finally, the solution of the original nonautonomous system (4) can be expressed through the solution of the averaged (autonomous) system (7) as follows:

8a $$\begin{aligned} v(t)&= \bar{v}(t)+A\psi (\omega t)+O(\omega ^{-1}), \end{aligned}$$

8b $$\begin{aligned} \mathbf{w}(t)&= \bar{\mathbf{w}}(t)+O(\omega ^{-1}). \end{aligned}$$

The substitution (2) and subsequent application of the averaging method allowed us to separate the slow and fast motion of the neuron and present the solution in the form of their superposition. The terms $\bar{v}(t)$ and $\bar{\mathbf{w}}(t)$ in Eq. (8) represent the slow motion and satisfy the averaged Eq. (7), while the term $A\psi (\omega t)$ describes the high-frequency oscillations of the membrane potential.

We emphasize that the averaged equations depend only on the parameter *A*, which is proportional to the ratio between the amplitude *a* and the frequency $\omega$ of the stimulating signal. This means that the effect of HFS on the neuron (more precisely, on its slow motion) is completely defined by this ratio. For example, the effect of HFS is the same if we fix the amplitude $a$ and double the frequency $\omega$ or fix the frequency $\omega$ and halve the amplitude $a$. Note that the dependence of the HFS effects on the ratio $a/\omega$ has been revealed in numerical simulations of the nerve conduction block, e.g., in Ref. Kilgore and Bhadra (2006), it was shown that for large $\omega$ the block threshold amplitude linearly depends on the HFS frequency.

In order to simplify the numerical simulation of the original equations, we perform the main analysis for the case of harmonic HF-stimulating signals, when $\varphi (\omega t) = \cos (\omega t)$ and $\psi (\tau )=\psi (\omega t)=\sin (\omega t)$. In the next section, we discuss the effect of nonharmonic charge-balanced stimulation.

## Results for the HF-stimulated HH neuron

To specify the details of the above approach, we first demonstrate it for the classical Hodgkin–Huxley model neuron (1952). Note, in the isolated HH neuron, periodic spiking does not emerge spontaneously [see e.g. Koch (1999)]. Rather, to induce a periodical spiking in the HH neuron, e.g., a constant current has to be injected. Where the oscillatory and synchronized activity in PD actually emerges is still a matter of debate. The most likely candidate generating these neuronal oscillations are the weakly interacting neuronal networks of the basal ganglia-thalamo-cortical loops (Bergman and Deuschl 2002). In the present study, we approximate the interaction by other neurons in a minimal model type of approach by injecting a current, which drives the single (otherwise noninteracting) model neuron. The frequency of the HH neuron has a well-defined nonzero minimum, which for standard parameters of the HH neuron typically exceeds the theta and beta frequency ranges relevant to PD (see e.g. Koch (1999), Sect. 6.4 therein). Accordingly, we do not use the HH model in order to model or mimic the Parkinsonian condition. Rather we use the HH model in order to illustrate our approach in a model that is mathematically relatively simple. The analysis of this model constitutes the basis for further application of the approximation based on the method of averaging to the more complex STN model (Terman et al. 2002), which will be considered in the next section. The HH model subject to HFS reads (Hodgkin and Huxley 1952):

9a $$\begin{aligned} C_m \dot{v}&= -I_L-I_{\mathrm{K}}-I_{\mathrm{Na}}+I_0+I_1 \cos (2\pi f t), \end{aligned}$$

9b $$\begin{aligned} \dot{m}&= \alpha _m (v) (1-m) -\beta _m (v) m, \end{aligned}$$

9c $$\begin{aligned} \dot{h}&= \alpha _h (v) (1-h) -\beta _h (v) h, \end{aligned}$$

9d $$\begin{aligned} \dot{n}&= \alpha _n (v) (1-n) -\beta _n (v) n. \end{aligned}$$

Here $C_m=1\,\upmu \hbox {F/cm}^2$ is the membrane capacitance, and $v$ is the membrane potential measured in mV. The leak $\hbox {Na}^+$ and $\hbox {K}^+$ currents are given by the following expressions

10a $$\begin{aligned} I_L&= g_{L}\left( v-v_L\right) \!, \end{aligned}$$

10b $$\begin{aligned} I_{\mathrm{K}}&= g_{\mathrm{K}}n^4\left( v-v_{\mathrm{K}}\right) \!, \end{aligned}$$

10c $$\begin{aligned} I_{\mathrm{Na}}&= g_{\mathrm{Na}} m^3 h(v-v_{\mathrm{Na}}). \end{aligned}$$

The parameters are as follows: $(v_L, v_{\mathrm{K}}, v_{\mathrm{Na}})=(10.6, -12, 115 )$ mV, ($g_L, g_{\mathrm{K}}, g_{\mathrm{Na}})=(0.3, 36, 120)\,\hbox {ms/cm}^2$. The rate parameters defining the dynamics of the gating variables $m,\,h$ and $n$ measured in $\hbox {ms}^{-1}$ are the following functions of the membrane potential:

11a $$\begin{aligned} \alpha _m(v)&= \left( 2.5-0.1 v\right) /\left[ \exp (2.5-0.1v)-1\right] , \end{aligned}$$

11b $$\begin{aligned} \beta _m(v)&= 4\exp (-v/18), \end{aligned}$$

11c $$\begin{aligned} \alpha _h(v)&= 0.07\exp (-v/20), \end{aligned}$$

11d $$\begin{aligned} \beta _h(v)&= 1/\left[ \exp (3-0.1v)+1\right] , \end{aligned}$$

11e $$\begin{aligned} \alpha _n(v)&= \left( 0.1-0.01v\right) /\left[ \exp (1-0.1v)-1\right] , \end{aligned}$$

11f $$\begin{aligned} \beta _n(v)&= 0.125\exp (-v/80). \end{aligned}$$

The parameters for this model have been obtained by fitting its solution to the experimental data on the giant axon of the squid (Hodgkin and Huxley 1952). Here, the voltage scale is shifted in such a way that the membrane resting potential (i.e., the steady-state value of the membrane potential without external currents, $I_0=I_1=0$) is zero.

**Fig. 1:**
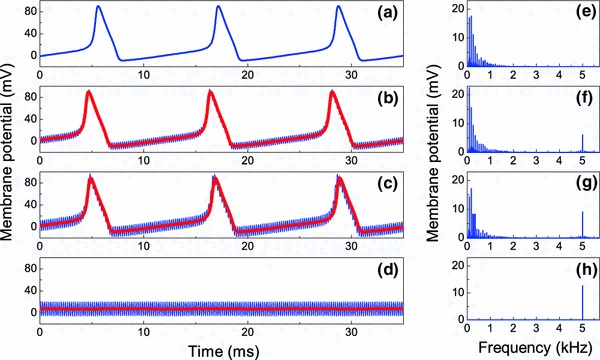
Influence of the high-frequency stimulation on the HH neuron dynamics (**a**)–(**d**) and spectrum (**e**)–(**h**). *Blue* and *red curves* represent the solutions of the original Hodgkin–Huxley model (9) and the averaged Eqs. (14), (16), respectively. Here, we show the post-transient dynamics for the initial conditions $(v,m,h,n)=(0,0,0,0)$. **a**, **e** $I_1=0$; **b**, **f** $I_1=200\,\upmu \hbox {A/cm}^2$; **c**, **g** $I_1=300\,\upmu \hbox {A/cm}^2$; **d**, **h** $I_1=400\,\upmu \hbox {A/cm}^2$

We apply a direct current $I_0=20\,\upmu \hbox {A/cm}^2$ in order to destabilize the resting state of the neuron and induce self-sustained periodic spiking. The dynamics of the membrane potential in the absence of stimulation ($I_1=0$) is shown in Fig. 1a. The neuron fires with the period $T \approx 11.57$ ms or characteristic frequency $\nu =1/T \approx 86.4$ Hz. The subsequent Fig. 1b–d shows the influence of charge-balanced HFS, which is modeled by the harmonic current $I_1 \cos (2\pi f t)$. When $f\gg \nu$, we can expect that the results given by the original HH model will coincide with those obtained from the averaged system. Here, we take the HFS frequency equal to $f=5$ kHz, which is typical for stimulation of peripheral neurons in order to produce a reversible block of undesired action potentials (Bhadra and Kilgore 2005; Bowman and McNeal 1986; Kilgore and Bhadra 2004, 2006; Williamson and Andrews 2005; Woo and Campbell 1964). Such stimulations, applied directly to the nerve, have been shown could help alleviate pain or stop muscle spasms (Long 1977; Nashold et al. 1982). In the next section, we will consider the STN model, whose characteristic spiking frequency without HFS is $\nu \approx 3$ Hz and the averaged system approximation for that model holds for considerably lower frequencies $f$ as for the HH model. Accordingly, the STN model will be analyzed in a frequency range relevant to DBS.

As seen from Fig. 1a–d, an increase in the simulation intensity $I_1$ from zero to 400 $\upmu \hbox {A/cm}^2$ induces drastic changes in the HH neuron dynamics. For small stimulation intensities, the low-frequency periodic spiking is only slightly modulated by the high-frequency oscillations. The increase in the stimulation intensity leads to an increase in the modulation amplitude. When the stimulation intensity reaches a certain threshold $I_1 \approx 379\,\upmu \hbox {A/cm}^2$, the neuronal sustained spiking suddenly disappears. In Fig. 1d, we see that for $I_1=400\,\upmu \hbox {A/cm}^2$, the membrane potential displays only high-frequency oscillations of moderate amplitude around a constant value close to the resting potential.

The effect of suppression of self-sustained spiking is particularly remarkable in the spectrum of the membrane potential shown in Fig. 1e–h. When the stimulation amplitude exceeds the threshold value, the low-frequency part of the spectrum related to the neuronal self-oscillations vanishes, and only a narrow, stimulation-related 5-kHz line remains.

To clarify the effect of suppression of low-frequency oscillations, we apply the technique described in the previous section. If the period of stimulation is much less than all characteristic times of the Hodgkin–Huxley neuron, an approximate solution of Eq. (9) can be presented in the form:

12a $$\begin{aligned} v(t)&\approx \bar{v}(t)+A \sin (2\pi f t), \end{aligned}$$

12b $$\begin{aligned} m(t)&\approx \bar{m}(t), \end{aligned}$$

12c $$\begin{aligned} h(t)&\approx \bar{h}(t), \end{aligned}$$

12d $$\begin{aligned} n(t)&\approx \bar{n}(t) , \end{aligned}$$

where

13 $$\begin{aligned} A=\frac{I_1}{2\pi f C_m} \end{aligned}$$

is the main parameter defining an action of the HFS. This parameter is proportional to the ratio of the amplitude $I_1$ to the frequency $f$ of HFS. Thus, the effect of HFS to the neuron dynamics is completely determined by this ratio. From Eq. (12), we see that the high-frequency “vibrational” component is added only to the membrane potential. The slow variables marked by bars describe the dynamics of the system averaged over the period of stimulation and satisfy the equations:

14a $$\begin{aligned} C_m \dot{\bar{v}}&= -g_l\left( \bar{v}-v_l\right) -g_{\mathrm{K}}\bar{n}^4\left( \bar{v}-v_{\mathrm{K}}\right) \nonumber \\&-g_{\mathrm{Na}} \bar{m}^3 \bar{h}\left( \bar{v}-v_{\mathrm{Na}}\right) +I_0, \end{aligned}$$

14b $$\begin{aligned} \dot{\bar{m}}&= \bar{\alpha }_m (\bar{v},A) (1-\bar{m}) -\bar{\beta }_m (\bar{v},A) \bar{m}, \end{aligned}$$

14c $$\begin{aligned} \dot{\bar{h}}&= \bar{\alpha }_h (\bar{v},A) (1-\bar{h}) -\bar{\beta }_h (\bar{v},A) \bar{h},\end{aligned}$$

14d $$\begin{aligned} \dot{\bar{n}}&= \bar{\alpha }_n (\bar{v},A) (1-\bar{n}) -\bar{\beta }_n (\bar{v},A) \bar{n}. \end{aligned}$$

Formally, these equations are similar to the original Eq. (9), but the HFS term is eliminated in Eq. (9a). The price one has to pay for this elimination is that the rate coefficients $\bar{\alpha }_X,\,\bar{\beta }_X\,=m,h,n)$ now depend not only on the membrane potential $\bar{v}$ but also on the stimulation parameter $A$. They are determined by averaging the original rate coefficients as follows:

15a $$\begin{aligned} \bar{\alpha }_X (\bar{v},A)=\frac{1}{2\pi } \int \limits _0^{2\pi } \alpha _X(\bar{v}+A \sin \tau ) \hbox {d}\tau , \end{aligned}$$

15b $$\begin{aligned} \bar{\beta }_X (\bar{v},A)=\frac{1}{2\pi } \int \limits _0^{2\pi } \beta _X(\bar{v}+A \sin \tau ) \hbox {d}\tau . \end{aligned}$$

If the parameter *A* is not very large, the averaged rate coefficients can analytically be estimated by the Taylor expansion

16a $$\begin{aligned} \bar{\alpha }_X (\bar{v},A)&\approx \alpha _X(\bar{v})+(1/4)A^2 \alpha ^{\prime \prime }_X(\bar{v}), \end{aligned}$$

16b $$\begin{aligned} \bar{\beta }_X (\bar{v},A)&\approx \beta _X(\bar{v})+(1/4)A^2 \beta ^{\prime \prime }_X(\bar{v}). \end{aligned}$$

Following the terminology of the vibrational mechanics, we refer to the terms in which $A$ appears [the last terms in Eq. (16)] as “vibrational forces.” The double-prime in these equations denotes the second derivative of a function. The vibrational forces are responsible for the changes in the slow component of the neuron dynamics induced by HFS. The solutions of the averaged equations shown in Fig. 1 by red curves are in good agreement with the solutions of the original Eq. (9) (blue curves).

Although Fig. 1a–h shows the effect of HFS for the fixed frequency $f$ and increasing amplitude $I_1$, the averaged equations tell us how this effect depends on the frequency $f$. Let us recall that the averaged dynamics is completely determined by the stimulation parameter $A$, which is proportional to the ratio $I_1/f$. Thus, for a fixed intensity $I_1$, a decrease in the stimulation frequency $f$ will cause a similar effect of suppression of neuron’s self-oscillations as shown in Fig. 1. Of course, this conclusion is only true for sufficiently large frequencies $f$, i.e., when $1/f$ is greater than the characteristic time scales of the neuron. If the frequency $f$ is not sufficiently large, the method of averaging fails, and HFS may cause more complicated effects than those presented in Fig. 1. In the next section, we discuss this issue for the STN neuron in more detail.

Now it is pertinent to discuss the question of how the results would change if the harmonic HFS were replaced by another charge-balanced waveform. In the case of harmonic HFS, the factor $(1/4)$ in the second terms of the Taylor expansion (16) results from averaging the square of the sinusoid $\left\langle \sin ^{2}(\tau )\right\rangle _{\tau } /2=(1/4)$. For an arbitrary waveform $\varphi (\tau )$, the factor (1/4) has to be replaced by the factor $\left\langle \psi ^{2}(\tau )\right\rangle _{\tau } /2$, where $\psi (\tau )=\int ^{\tau } \varphi (s) ds$. The variation in the above factor can be compensated by rescaling the parameter $A$. Thus, if the expansion (16) is valid, the variation in the waveform has no principal effect on the final results except for rescaling the stimulation parameter $A$. We have verified this conclusion numerically for several different waveforms of HFS including those typically used for DBS and obtained a significant agreement between theory and numerics (results not presented here).

**Fig. 2:**
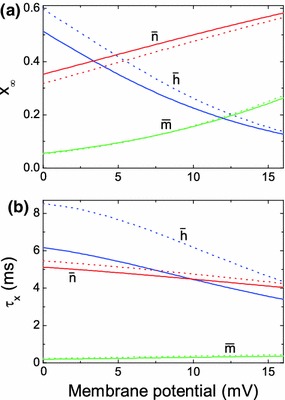
Dynamical properties of the averaged HH neuron equations. **a**, **b** the dependence of $x_\infty (\bar{v}, A)$ and $\tau _x(\bar{v}, A)$ on the averaged membrane potential $\bar{v}$, respectively. Here $x$ stands for $\bar{m}$ (*green*), $\bar{h}$ (*blue*) or $\bar{n}$ (*red*) gating variables. The *dotted lines* show the dependencies without stimulation ($A=0$), and the *solid lines* correspond to $A=17$ mV

Note that the vibrational forces appear only in Eqs. (14b)–(14d) that govern the dynamics of the gating variables. Hence, HFS exclusively acts on the ion channels. In order to get a better understanding of how the vibrational forces modify the neuron dynamics, we rewrite each of the Eqs. (14b)–(14d) in the form

17 $$\begin{aligned} \dot{x}=-\frac{1}{\tau _x(\bar{v}, A)}\left[ x-x_\infty (\bar{v}, A)\right] , \end{aligned}$$

where $x$ stands for $\bar{m},\,\bar{h}$ or $\bar{n}$. For fixed $\bar{v}$ and $A$, the variable $x$ approaches the value $x_\infty (\bar{v}, A)$ with time constant $\tau _x(\bar{v}, A)$. The asymptotic value $x_\infty (\bar{v}, A)$ and the time constant $\tau _x(\bar{v}, A)$ are given by the transformation $x_\infty (\bar{v}, A) = \bar{\alpha }_x(\bar{v}, A)/[\bar{\alpha }_x(\bar{v}, A)+\bar{\beta }_x(\bar{v}, A)]$ and $\tau _x(\bar{v}, A)=1/[\bar{\alpha }_x(\bar{v}, A)+\bar{\beta }_x(\bar{v}, A)]$. The variables $x_\infty$ and $\tau _x$ are plotted as functions of $\bar{v}$ for two different values of $A$ in Fig. 2a, b, respectively. The HFS has almost no influence on the sodium activation variable; the functions $\bar{m}_\infty (\bar{v}, A)$ and $\tau _m(\bar{v}, A)$ change only slightly when $A$ is increased from 0 to 17 mV. However, the stimulation influences considerably the sodium inactivation and potassium activation processes. The stimulation decreases $\bar{h}_\infty (\bar{v}, A)$ and increases $\bar{n}_\infty (\bar{v}, A)$. Both effects are inhibitory and may lead to a depolarization block, because the conductance of the intracellular sodium current tends to decrease to the value $g_{Na}\bar{m}_\infty ^3(\bar{v}, A)\bar{h}_\infty (\bar{v}, A)$, while the conductance of the extracellular potassium current tends to rise to the value $g_k\bar{n}_\infty ^4(\bar{v}, A)$. In addition, these inhibitory tendencies are accelerated by the HFS, since both $\tau _h(\bar{v}, A)$ and $\tau _n(\bar{v}, A)$ decrease as $A$ increases.

**Fig. 3:**
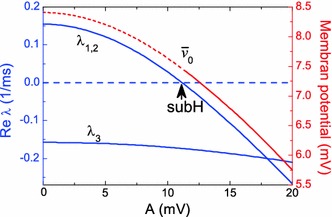
The real parts of the eigenvalues of the steady-state solution of the averaged HH Eq. (14) as functions of the stimulation parameter $A$. Here, $\lambda _{1,2}$ marks a complex–conjugate pair of leading eigenvalues, $\lambda _3$ is a real negative eigenvalue. The smallest negative eigenvalue $\lambda _4$ is not shown in the diagram. The symbol “subH” denotes the point of the subcritical Hopf bifurcation. The *red line* shows the dependence of the resting potential $\bar{v}_0$ on $A$. The unstable part is depicted by the *dashed line*

In Fig. 3, we plot the steady-state solution $\bar{v}_0$ of the averaged Eq. (14) and the eigenvalues of this solution as functions of the stimulation parameter A. The resting potential $\bar{v}_0$ decreases with the increase in A, and for a certain threshold $A=A_{\mathrm{subH}}\approx 11.16$ mV, the resting state becomes stable through a subcritical Hopf bifurcation. The stabilization of the resting state explains the death of the low-frequency oscillations presented in Fig. 1. We emphasize that the stability of the resting state of the averaged equations does not mean that the membrane potential $v(t)$ is independent of time. This stabilization means that only the slow component of the membrane potential is constant, $\bar{v}(t)=\bar{v}_0$. According to Eq. (12a), the total value of the membrane potential consists of a sum of slow and fast components $v(t)=\bar{v}_0+A \sin (2\pi f t)$, and thus, it experiences HF oscillations around the resting state $\bar{v}_0$ with the amplitude $A$ and frequency $f$. Note that the stabilization of the aforementioned Kapitsa’s pendulum manifests itself in a similar way. When the pendulum is stabilized in the upside-down position, it still experiences small high-frequency oscillations around the stabilized equilibrium point.

**Fig. 4:**
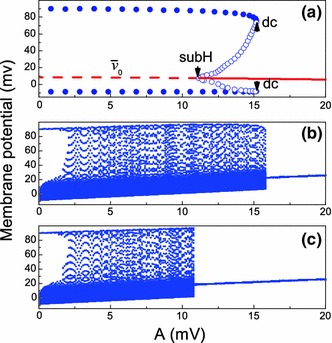
Bifurcation diagrams of the HH neuron under HFS. **a** the bifurcation diagram obtained from the averaged Eqs. (14), (16). The *red line* shows the resting potential $\bar{v}_0$, i.e., the same curve, which is presented in Fig. 3, at an extended scale. The unstable part is depicted by a *dashed line*. The *solid dots* and *open circles* represent the stable and unstable limit cycles, respectively. The symbol “subH” denotes the point of the subcritical Hopf bifurcation, and “dc” marks the double-cycle bifurcation. **b**, **c** the maxima of the membrane potential obtained from the original Eq. (9) for increasing and decreasing values of the stimulation parameter $A=I_1/(2\pi f C_m)$, respectively. The *horizontal axis* displays the values of the parameter $A$ for fixed $f=5$ kHz and varying $I_1$

The results of a global phase space analysis of the averaged equations are summarized in the bifurcation diagram shown in Fig. 4a. When varying the stimulation parameter $A$, the system experiences jumps and hysteresis. For $A = 0$, there is an unstable fixed point and the stable limit cycle is responsible for the neuron sustained spiking. When $A$ is increased to the value $A_{\mathrm{dc}} \approx 15.17$ mV, a double-cycle bifurcation (the point at which the stable limit cycle collides with an unstable limit cycle) takes place, and the system jumps to a stable fixed point. This explains the death of self-oscillations. If we now decrease $A$, the system remains in the stable resting state up to the value $A = A_{\mathrm{subH}}$ and then jumps back to the stable limit cycle. In the interval $A_{\mathrm{subH}}< A < A_{\mathrm{dc}}$, the system is bistable; depending on initial conditions, it may approach either the stable fixed point or the stable limit cycle.

In Fig. 4b, c, we show the bifurcation diagrams (the maxima of the membrane potential $v$) obtained from the original HH equations for increasing and decreasing values of the stimulation parameter $A$. Comparing these results with those presented in Fig. 4a, we can conclude that the averaged equations correctly predict and explain the bifurcations and the hysteresis observed in the original HH equations. We see that the jumps of the amplitude of the membrane potential are related to the subcritical Hopf and double-cycle bifurcations in the averaged equations. The small amplitudes of the membrane potential correspond to a stable resting state of the averaged dynamics.

## Results for the HF-stimulated STN model neuron

To apply the above ideas to DBS in PD, we consider an STN model neuron (Terman et al. 2002), which represents a modified version of the HH model adapted to the physiology of the STN. The STN is a major target for HF DBS in PD patients (Limousin et al. 1995, 1998; Rodriguez-Oroz et al. 2000). The equation for the membrane potential of an STN neuron under HFS reads:

$$\begin{aligned} C_m \dot{v} \!=\! -I_L\!-\!I_{\mathrm{K}}-I_{\mathrm{Na}}-I_T-I_{\mathrm{Ca}}-I_{\mathrm{AHP}}+I_1 \cos (2\pi f t). \end{aligned}$$

The currents $I_L=g_L(v-v_L),\,I_{\mathrm{K}}=g_{\mathrm{K}}n^4(v-v_{\mathrm{K}})$ have the same expressions as for the HH model, while $I_{\mathrm{Na}}=g_{\mathrm{Na}}m_{\infty }^3(v) h(v-v_{\mathrm{Na}})$. The additional currents $I_T= g_T a_{\infty }^3(v) b_{\infty }^3(r)(v-v_{\mathrm{Ca}}),\,I_{\mathrm{Ca}}= g_{\mathrm{Ca}}s_{\infty }^2(v)(v-v_{\mathrm{Ca}})$ and $I_{\mathrm{AHP}}=g_{\mathrm{AHP}}(v-v_\mathrm{K})([\mathrm{Ca}]/([\mathrm{Ca}]+k_1))$ are related to the dynamics of the $\mathrm{Ca}^{2+}$ ions. The gating variables $n,\,h$, and $r$ are governed by differential equations of the form $\dot{X}=\phi _X(X_\infty (v)-X)/\tau _X(v)$ (where $X$ can be $n,\,h$ or $r$), with $\tau _X(v)=\tau _X^0+\tau _X^1[1+\exp [-(v-\theta _X^\tau )/\sigma _X]]$. Activation gating for the channels $m,\,a$, and $s$ are treated as instantaneous. For all gating variables $X=n,\,m,\,h,\,a,\,r$ or $s$, the steady state is determined by $X_\infty (v)=1/[1+\exp [-(v-\theta _X)/\sigma _X]]$. The activation variable $b$ is defined by $b_\infty (r)=1/[1+\exp [(r-\theta _b)/\sigma _b]]-1/[1+\exp (-\theta _b/\sigma _b)]$. Finally, the intracellular concentration $[\mathrm{Ca}]$ of the $\mathrm{Ca}^{2+}$ ions is governed by $\dot{[\mathrm{Ca}]}=\epsilon (-I_{\mathrm{Ca}}-I_T-k_{\mathrm{Ca}}[\mathrm{Ca}])$. The values of the parameters are presented in Reference Terman et al. (2002).

**Fig. 5:**
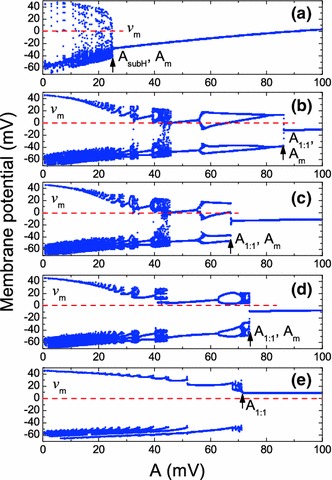
Bifurcation diagrams of the STN neuron under HFS. The local maxima of the membrane potential as a function of the stimulation parameter $A=I_1/2\pi f C_m$ are shown for varying $I_1$ and fixed $f$: **a** $f=3$ kHz; **b** and **c** $f=200$ Hz; **d** $f=150$ Hz; **e** $f=60$ Hz. The bifurcation diagrams (**a**), (**b**), (**d**), and (**e**) are computed for the increasing $I_1$, while (**c**) is obtained for the decreasing $I_1$. The *red dash lines* show the threshold voltage $v_m$

The free ($I_1=0$) STN neuron exhibits self-sustained spiking around the unstable resting state $v_0\approx -37.78$ mV with amplitude $\approx 45.2$ mV and frequency $\approx 2.7$ Hz. Contrary to the HH model, here the spiking activity emerges spontaneously without direct current. Bifurcation diagrams in Fig. 5 illustrate the influence of HFS on the neuron dynamics for different values of the frequency $f$. For sufficiently large *f* ($>600$ Hz), the death of the neuron’s self-oscillations can again be explained by a stabilization of the resting state. The fixed point of the averaged STN equations becomes stable through a subcritical Hopf bifurcation at $A_{\mathrm{subH}} \approx 24.12$ mV. This value provides a correct prediction for the jump of the amplitude of the oscillations observed in Fig. 5a. Note that for the averaged STN equations, the hysteresis is less pronounced than for the corresponding HH equations. Hence, in Fig. 5a, we have restricted ourselves to present the evolution of the dynamics only for growing values of $A$.

If the frequency $f$ is not large (i.e., $1/f$ is comparable to the characteristic neuronal time scales), the averaged equations are not valid. As shown in Fig. 5b–e, in this case, the system exhibits complex bifurcations. A significant hysteresis is observed for an intermediate frequency interval $160<f<350$ Hz. An example of such a hysteresis for a fixed frequency $f=200$ Hz is demonstrated in Fig. 5b, c. The different bifurcation scenarios are observed for the increasing (b) and decreasing (c) stimulation intensity $I_1$. For lower frequencies, $f<160$ Hz, the hysteresis disappears. The bifurcation diagrams in Fig. 5d, e corresponding to the fixed frequencies $f=150$ and $f=60$ Hz, respectively, are presented only for the growing $I_1$.

Although the averaged equations for low frequencies are not valid and we cannot interpret the results presented in Fig. 5b–e in terms of a stabilization of the fixed point of the averaged neuron dynamics, the pronounced jump of the oscillation amplitude is still present in these figures. This jump appears at a transition to $1:1$ synchronization. We denote the critical value of $A$ corresponding to this transition by $A_{1:1}$. Note that in the case of HF (when the averaged equations are valid), the jump of the oscillation amplitude associated with the stabilization of the neuron resting state can be also explained in terms of $1:1$ synchronization. Indeed, in the averaging approach, the total neuron dynamics is defined by the sum of slow and fast components, $v(t)=\bar{v}(t)+A \sin (2\pi ft)$. The frequency of the fast component coincides with the frequency of the stimulation signal. When the fixed point is stabilized by HFS, then the slow component becomes constant, $\bar{v}(t)=\hbox {const}$. But still there always remains the high-frequency component, whose frequency is in $1:1$ relation with the stimulation signal. We emphasize that these high-frequency oscillations represent the harmonic signal, i.e., they have the same profile as the stimulation signal and differ considerably from the profile of the action potential of the free neuron. Moreover, the amplitude of these oscillations is small, and thus, they cannot be interpreted as spikes of the action potential induced by HFS. Nevertheless, one can say that the concept of $1:1$ synchronization is more general than the concept of the stabilization of the fixed point in the averaging approach, since formally it can be used for any frequencies of the stimulation signal. If the stimulation frequency is comparable with the eigenfrequency of the neuron, the jump of the oscillation amplitude can be explained in terms of $1:1$ synchronization, but cannot be interpreted in terms of a stabilization of the fixed point of averaged dynamics. When the stimulation frequency is considerably higher than the eigenfrequency of the neuron, the description of the regime of $1:1$ synchronization can be mathematically simplified and interpreted in terms of a stable fixed point of the averaged equations plus high-frequency harmonic oscillations.

An alternative way to characterize the suppression of neuronal oscillations is to introduce a threshold amplitude $v_m$ of the membrane potential at which the neuronal oscillations can be disregarded. We can treat $v_m$ as a minimal amplitude of the STN neuron necessary to excite a postsynaptic globus pallidus external (GPe) neuron. Then by $A_m$, we denote the characteristic value of $A$ at which the STN neuron amplitude falls below $v_m$. To estimate the threshold amplitude $v_m$, we evaluate the synaptic current flowing from the STN to the GPe neuron. According to Reference Terman et al. (2002), the strength of this current is defined by the synaptic variable $S$ that satisfies an equation

18 $$\begin{aligned} \dot{S}=\left[ S_\infty (v)-S\right] /\tau _S(v). \end{aligned}$$

The function $S_\infty (v)$ represents a sigmoid curve with a characteristic threshold voltage approximately equal to 0 mV [this estimation is based on the parameter values presented in Terman et al. (2002)]. We interpret this threshold voltage as a minimal amplitude of the STN neuron necessary to excite the GPe neuron, i.e., we take $v_m=0$ mV. The threshold value $v_m$ cannot be defined in a rigorous way and is to some extent arbitrary. The chosen value $v_m$ = 0 mV provides a good fit of our model to the experimental data. The analysis of the bifurcation diagram for low frequencies reveals that there exists a critical frequency $f_c \approx 95$ Hz such that for $f<f_c$, the amplitude of the neuronal oscillations exceeds the threshold value $v_m$ at any $A$ [cf. Fig. 5e]. Below, we will show how the results based on the defined threshold value correlate with the results of the direct simulation of Eq. (18).

**Fig. 6:**
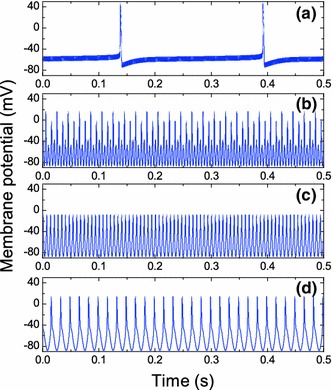
STN neuron dynamics for a fixed stimulation amplitude $I_1=8$ mA/cm$^2$ and different stimulation frequencies: a $f=3$ kHz; **b** $f=200$ Hz; **c** $f=150$ Hz; **d** $f=60$ Hz

The STN neuron dynamics for a fixed stimulation amplitude $I_1=8\,\hbox {mA/cm}^2$ and different stimulation frequencies is demonstrated in Fig. 6. For a high-frequency $f=3$ kHz, the stimulation intensity $I_1=8\,\hbox {mA/cm}^2$ is not sufficient to stabilize the neuron’s resting state and we observe a self-sustained neuronal spiking of rather high amplitude slightly modulated with the HFS signal (Fig. 6a). For the frequency $f=200$ Hz, we observe the regime of $1:4$ synchronization whose absolute maxima exceed the threshold amplitude $v_m=0$ mV (Fig. 6b). In Fig. 6c, the neuron’s dynamics is presented for a typical therapeutic frequency $f=150$ Hz. Here, we observe the subthreshold tonic $1:1$ oscillations. These oscillations represent almost a harmonic signal, i.e., their profile coincides with the profile of the stimulation signal. A further decrease in the stimulation frequency leads to HFS-induced $1:1$ spiking regime with spike amplitude exceeding the threshold value $v_m=0$ mV. This is demonstrated in Fig. 6d for the frequency $f=60$ Hz. Now the neuron dynamics differs considerably from the harmonic signal and resembles the action potential of the free STN neuron. In fact, the transition to the HFS-induced spiking regime explains why the maxima of the membrane potential increase with the decrease in the stimulation frequency.

**Fig. 7:**
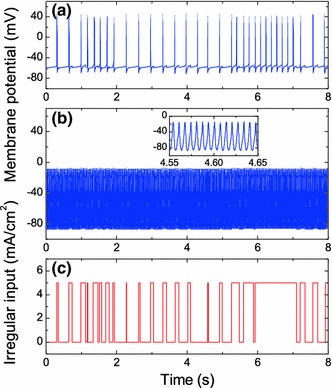
Suppression of the STN spiking by HFS in the presence of an additional irregular input current. **a** Dynamics of the membrane potential under irregular input but without HFS. **b** Dynamics of the membrane potential in the presence of both irregular current and HFS with $I_1=8\,\hbox {mA/cm}^2$ and $f=150$ Hz. The *inset* shows the potential trace on an expanded scale. **c** The dynamics of the irregular input current delivered to the membrane. This current has been generated by the Heaviside function whose argument represents a sum of harmonic signals with different incommensurable frequencies

Subthalamic nucleus neurons in vivo are subjected to a barrage of synaptic inputs that cause them to spike in a manner which cannot be considered as strictly periodic, even under Parkinsonian conditions where spiking is more regular than in healthy states. In Fig. 7, we present the results of simulations of the STN neuron in the presence of an additional irregular input current delivered to the membrane [diagram (c)], which causes an irregular spiking of the STN [diagram (a)]. Diagram (b) shows that the HFS with the intensity $I_1=8\,\hbox {mA/cm}^2$ and the frequency $f=150$ Hz efficiently suppresses the irregular spiking of the STN in this case as well. Here as well as in Fig. 6c, we observe tonic 1:1 subthreshold oscillations. As shown in the inset of panel (b), the shape of these oscillations differs considerably from the action potential of the free STN neuron and almost coincides with the shape of stimulating (harmonic) signal. In terms of the averaging approach, this state can be interpreted as a sum of a stabilized resting state of a slow dynamics and a fast high-frequency component directly induced by the HFS. An additional irregular current is unable to excite the neuron from the stabilized resting state and has almost no influence on the final neuron’s dynamics.

**Fig. 8:**
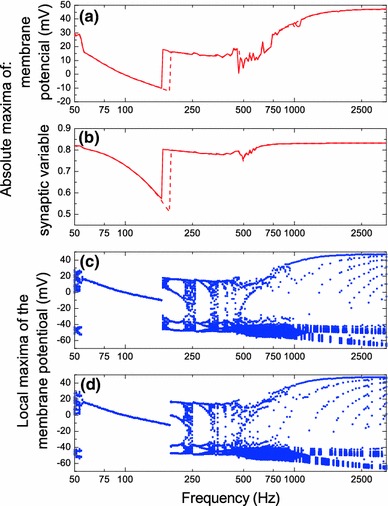
Absolute maxima of the membrane potential (**a**) and the synaptic variable $S$ (**b**) of the STN neuron as functions of the stimulation frequency for a fixed stimulation amplitude $I_1=8\,\hbox {mA/cm}^2$. The parameters for Eq. (18) defining the dynamics of the synaptic variable $S$ are taken from Ref. Terman et al. (2002). The *solid* and *dashed curves* refer to increasing and decreasing stimulation intensity, respectively. **c**, **d** The local maxima of the membrane potential for increasing and decreasing stimulation intensity, respectively

A more detailed amplitude–frequency characteristic of the STN neuron for a fixed stimulation intensity $I_1=8\,\hbox {mA/cm}^2$ is presented in Fig. 8. In the diagram (a), we show the absolute maxima (unlike to the local maxima shown in the bifurcation diagrams of Fig. 5) of the membrane potential in dependence of the stimulation frequency. The solid and dashed curves correspond to increasing and decreasing stimulation intensity, respectively. Diagram (b) shows the absolute maxima of the synaptic variable *S* obtained by direct simulation of Eq. (18). For a given stimulation intensity $I_1=8\,\hbox {mA/cm}^2$, our model predicts that in the frequency interval $97\,\hbox {Hz}<f<170$ Hz, the maxima of the synaptic variable S reduce noticeably, and the maxima of the membrane potential fall below the threshold value $v_m=0$ mV. In order to demonstrate how these results are related to different synchronization regimes (1:1 or higher order) in (c) and (d), we present the standard bifurcation diagrams, which show the local maxima of the membrane potential in dependence of the stimulation frequency.

**Fig. 9:**
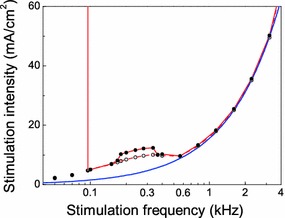
Two-parametrical bifurcation diagram of a HF stimulated STN neuron: comparison between theory (*blue curve*) and direct numerical simulations (*red curves*). The *red curves* show the critical current $I_1$ when the amplitude of oscillations reaches the threshold voltage $v_m=0$ mV. The *solid* and *dashed red curves* refer to increasing and decreasing stimulation intensity, respectively. They only slightly differ in a small interval of intermediate frequencies. The *solid dots* and *open circles* show the threshold of $1:1$ synchronization for increasing and decreasing stimulation intensity, respectively. At the left-hand side of the *red vertical line*, the amplitude of the neuronal oscillations exceeds the threshold value $v_m$ for any $I_1$. The *blue curve* $I_1=2\pi f C_m A_{\mathrm{subH}}$ shows the subcritical Hopf bifurcation of the averaged STN equations. The deviation of the *red curves* from the *blue curve* for frequencies below 600 Hz is due to the decreased accuracy of the averaged equations for low frequencies

Bradykinesia, rigidity, and tremor are three major PD symptoms [see e.g. Rivlin-Etzion et al. (2006)]. The neuropathologic basis of PD with predominant resting tremor significantly differs from that of PD with marked bradykinesia and rigidity (Paulus and Jellinger 1991). STN DBS is used in PD patients either who suffer from bradykinesia and rigidity only or who suffer from all three symptoms (bradykinesia, rigidity, and tremor) (Limousin et al. 1995, 1998; Rodriguez-Oroz et al. 2000). Clinical data suggest that the mechanism by which STN DBS acts on tremor significantly differs from that on bradykinesia and rigidity (Temperli et al. 2003): After turning off clinically effective STN DBS, tremor reoccurs within minutes, whereas bradykinesia and rigidity reemerge within half an hour to an hour. We here focus on the effect of DBS on tremor. This is because electrophysiological studies in PD patients indicate a causal relationship between tremor and the local field potential in the STN or the ventro-intermediate (VIM), respectively (Tass et al. 2010). Also, in akinetic patients, the effect of DBS is more difficult to quantify (Moro et al. 2002). We have chosen an STN model neuron for our analysis, since the STN is a major target for DBS in PD (Limousin et al. 1995, 1998; Rodriguez-Oroz et al. 2000), and for the STN, there are established neuronal models available (Gillies and Willshaw 2004; Modolo et al. 2008; Terman et al. 2002). Moro et al. (2002) analyzed the effects of STN DBS at different stimulation frequencies (50, 130, 185, and 250 Hz) on tremor, bradykinesia, and rigidity, thereby keeping amplitude and pulse width constant. Stimulation frequencies above 50 Hz caused a significant relief of the three symptoms, where a maximum benefit was achieved at 185 Hz. Differences in improvement between 50 and 130 Hz or 50 and 185 Hz were significant. Statistical analysis of the effect on tremor for the stimulation frequency 250 Hz was impossible due to a limited number of patients treated with this frequency. So, from a statistical point of view, we can only compare the tremor-suppressing effect at 50, 130, and 185 Hz. While at 50 Hz, there is no tremor suppression, at 130 and 185 Hz, tremor is effectively suppressed. This is in complete agreement with our results: In Fig. 9, the analysis of the STN model is summarized in a two-parametrical bifurcation diagram with stimulation frequency $f$ and intensity $I_1$ as control parameters. The red curve shows the critical current $I_1$ at which the neuron amplitude falls below the threshold value $v_m$. More detailed experimental data are available for DBS of the VIM nucleus of the thalamus (Benabid et al. 1991), which is used in tremor-dominant PD patients. Intriguingly, the dependence revealed by our analysis (Fig. 9) resembles the relation between stimulation frequency and intensity necessary to abolish tremor in PD patients experimentally obtained for VIM DBS (Benabid et al. 1991). The interval of characteristic frequencies confined by the red curve is comparable with that where the therapeutic effect was observed in the clinical experiments (Benabid et al. 1991). Note, in the STN model, neuron hysteresis, i.e., a different bifurcation behavior depending on whether stimulation intensity increases (solid red curve) or decrease (dashed red curve), is only present in a confined interval of intermediate frequencies.

The blue curve in Fig. 9 represents the curve of the subcritical Hopf bifurcation determined from the averaged STN neuron equations. For HF, it coincides with the red curve. Thus, the suppression of oscillations for HF can be interpreted as a stabilization of the neuron’s resting state. The comparison between theory (i.e., averaged equations, blue curve in Fig. 9) and direct numerical simulations (red curves in Fig. 9) shows that the accuracy of the averaged equations is limited for frequencies below 600 Hz.

Finally, we have estimated the current density injected into STN neurons by a DBS electrode. Generally, this is a rather complex problem, which depends on many factors including the geometry of the electrode as well as the distance and orientation of axons with respect to the electrode. The second derivative of the extracellular potential distribution along a neural process $(\partial ^2 V/\partial x^2)$ provides a quantitative estimate of the injected current density $I$ in response to an applied electric field (Rattay 1989):

19 $$\begin{aligned} I(x,t)=\frac{\hbox {d} \varDelta x}{4 \rho _i L}\frac{\partial ^2 V(x,t)}{\partial x^2}. \end{aligned}$$

**Fig. 10:**
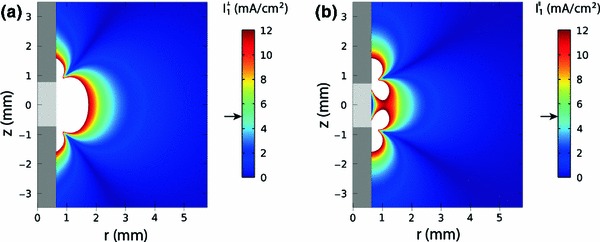
Distribution of the amplitude of injected current density for STN axons orientated perpendicular (**a**) and parallel (**b**) to the DBS electrode in the $(z, r)$ plane of the cylindrical coordinate system. The DBS contact (*light gray color*) is represented by a cylinder of 1.27 mm in diameter and 1.5 mm in height. The insulator is shown by *dark gray color*. The calculations are performed for a HFS amplitude equal to 3 V. The spatial distribution of the amplitude of injected current density is represented by colors according to the *color bar* at the right. The current densities exceeding the maximal value of the *color bar* (12 mA/cm$^2$) are represented by *white color*. The *arrow* at the *color bar* shows the color corresponding to the threshold density 5 mA/cm$^2$. The current densities are estimated from Eq. (19) using the second derivative of the potential $(\partial ^2 V/\partial r^2)$ and $(\partial ^2 V/\partial z^2)$ for axons orientated perpendicular and parallel to the DBS electrode, respectively. The parameters of the STN axon are (Sotiropoulos and Steinmetz 2007; Grill et al. 2008): $d=2\,\upmu \hbox {m}, L=1\,\upmu \hbox {m}, \Delta x=200\,\upmu \hbox {m}$ and $\rho _i=70\,\Omega$cm

Here $d$ is the axon diameter, $\rho _i$ is the resistivity of the axoplasm, $L$ is the active length of the membrane (node length), and $\Delta x$ is the segmentation length (node–node separation) of a myelinated axon. To estimate the spatial distribution of the extracellular potential $V$, we have solved the 3D Laplace’s equation $\Delta V=0$ with appropriate boundary conditions. The calculations were performed with the COMSOL Multiphysics 4.0a package for a clinical DBS electrode with a cylindrical contact of 1.27 mm in diameter and 1.5 mm in height. We have considered a monopolar stimulation and used a clinically typical value of the HFS voltage amplitude equal to 3 V. The distributions of injected current densities for STN axons orientated perpendicular and parallel to the DBS electrode are shown in Figs. 10a, b, respectively. According to the results presented in Fig. 9, our model predicts the suppression of spontaneous firing when the current density exceeds a threshold value equal to 5 $\hbox {mA/cm}^2$. As seen from Fig. 10, the domain of suppressed spontaneous neuronal firing (where the amplitude of injected current exceeds the threshold value 5 mA/cm$^2$) is rather large; the effect takes place in a region of 1–2 mm in the vicinity of the electrode. Thus the described mechanism of suppression of STN spontaneous firing is realistic for a typical clinical setting.

## Discussion

For frequencies of HFS considerably greater than the reciprocal of the neuron’s characteristic time, the system dynamics can be separated into fast and slow components. The slow component satisfies an autonomous system of averaged equations, which depends on only one parameter, which is proportional to the ratio between the amplitude and the frequency of the stimulating signal. We have shown that the suppression of the self-sustained spiking by HFS is related to the stabilization of the neuron’s resting state of the averaged dynamics. This fundamental mechanism has an analogy to nonlinear mechanical systems, where the vibration may cause a stabilization of unstable equilibrium points. For the STN neuron model, this mechanism qualitatively reproduces the clinically measured relationship between stimulation frequency and intensity necessary to suppress tremor in patients with PD.

Terman et al. (2002) extended the original HH model (1952), comprising equations for sodium, potassium, and leak currents, by introducing three different additional currents: a low-threshold $\hbox {Ca}^{2+}$ current, a high-threshold $\hbox {Ca}^{2+}$ current as well as a voltage-independent $\hbox {Ca}^{2+}$-governed potassium current. This implies an increase in dimensionality and more dynamical complexity in the STN neuron model, especially due to the slow $\hbox {Ca}^{2+}$ dynamics. Nevertheless, HFS stops regular spiking in both the HH and STN neuron models. In fact, in the present study, we showed that in single HH or STN model neurons HFS stops regular spiking by stabilization of the neuron’s resting state or by stabilization of a low-amplitude subthreshold oscillation of its membrane potential. In the context of DBS, from an experimental standpoint, it is not trivial to measure the membrane potential during stimulation (e.g., due to stimulation artifacts) and to determine whether a single neuron is actually silent or displays low-amplitude subthreshold membrane potential oscillations. Still, there are a number of studies that enable us to compare our theoretical results with experimental findings. The inhibitory effect of HFS revealed here is completely compatible with the observation that the clinical effects induced by lesions and DBS of the same target area are similar (Limousin et al. 1995). Also, several experiments in vitro (Beurrier et al. 2001; Magariños-Ascone et al. 2002), in animals (Benazzouz et al. 2000; Moran et al. 2011; Tai et al. 2003) and in humans (Filali et al. 2004; Welter et al. 2004), support the hypothesis of a locally inhibiting effect of HF DBS. In rat STN slices in vitro Beurrier et al. (2001) showed that brief high-frequency pulse trains at a frequency in the range of 100–250 Hz delivered during 1 min caused a full blockade of ongoing STN activity, in both tonic and bursting mode. The HFS-induced blockade lasted up to 6 min after cessation of stimulation and was not synaptically induced, since it was still present when inotropic GABA and glutamate receptors or voltage-gated $\hbox {Ca}^{2+}$ channels were blocked (Beurrier et al. 2001). The full blockade during HFS observed by Beurrier et al. (2001) is in perfect agreement with our theoretical results presented here. The observation that the blockade temporarily outlasts cessation of HFS is a phenomenon one would not necessarily expect based on our results, obtained in an isolated neuron. This effect might result in plasticity in the synaptic coupling between neurons within the network that are not explicitly modeled here. Still, this aspect remains to be clarified in a future study employing a more complex, network type of model incorporating plasticity effects. In monkeys, rendered Parkinsonian with the neurotoxin MPTP (Moran et al. 2011) performed STN high-frequency macro-stimulation. Simultaneously, they recorded in the STN and in the globus pallidus externus and internus. The neurons responses to HFS were stereotypical within the different nuclei and differed between the nuclei. HFS predominately caused a somatic inhibition of STN neurons, which is in accordance with our findings. In the majority of pallidal neurons, HFS gave rise to somatic activation. Axonal activation was found in only a minority of neurons across all nuclei. Of course, the latter effects cannot be reproduced by our minimal STN single neuron model.

In urethane-anaesthetized rats, STN stimulation at 130 Hz with pulse width 60 ms, intensities 10–1,000 $\upmu$A, and train duration 5 s caused a massive decrease in the firing rate of STN neurons, which after cessation of stimulation continuously re-increased to the pre-stimulus level (Benazzouz et al. 2000). A likely explanation of this effect is that STN HFS induces a depolarization block (Benazzouz et al. 2000), especially because of the characteristic responses of neurons in the substantia nigra pars reticulata (SNr) to STN stimulation (Benazzouz et al. 2000, 1995): SNr firing is increased by single shocks and by low-frequency stimulation, whereas HFS causes a strong decrease in SNr activity. However, since during STN HFS recordings in the STN were not feasible (due to artifacts), Benazzouz et al. (2000) could not strictly rule out other possible effects of STN HFS on STN neurons, such as disruption of one or more neural networks, achievement of a net inhibitory effect due to preferential activation of inhibitory neurons, or a network-related rather than single neuron-related response to HFS. The depolarization block hypotheses are in perfect agreement with our theoretical study, whereas the other potential mechanisms refer to mechanisms beyond a single neuron and, hence, cannot be captured with the single neuron approach presented here.

In twelve PD patients, the effects of STN HFS were studied during stereotactic procedures for implantation of depth electrodes for DBS (Filali et al. 2004). Neural activity of STN cells was recorded with one electrode, while stimulation was delivered through a second electrode located approx. 600 $\upmu$m apart. HF STN stimulation at HF (100–300 Hz) caused an inhibition following the high-frequency pulse train in 42 % of the 60 cells tested (Filali et al. 2004). In 15 neurons, it was possible to study stimulation effects during delivery of HFS. In 13 out of these 15 cells, Filali et al. (2004) observed inhibition. In 44 % of the neurons where HFS was inhibitory, after cessation of stimulation and early inhibitory phase was followed by rebound excitation and a further inhibitory period. This was interpreted as being indicative of HFS causing a hyperpolarization (Filali et al. 2004). The inhibitory action of HFS as well as the HFS-induced hyperpolarization reported and discussed by Filali et al. (2004) are in line with our theoretical findings on the HFS-induced stabilization of low-threshold (hyperpolarized) oscillations. However, we did not study after-effects. The latter will presumably be strongly influenced by the network topology.

Single-unit recordings of STN activity were performed in 15 PD patients during depth electrode implantation by Welter and coworkers (Welter et al. 2004). To this end, stimulation at different frequencies (14, 40, 80, and 140 Hz) was delivered via one electrode, whereas recording was performed through another electrode. Single-unit activity was recorded 20 s before, during, and after stimulation. Stimulation at frequencies $>$40 Hz caused a decrease in the firing frequency, partially even a complete arrest, and an increase in the burst-like activity in the STN cells (Welter et al. 2004). An inhibitory after-effect was observed in neurons that had been totally inhibited by HFS (Welter et al. 2004). Both the HFS-induced decrease in the firing rate and the complete blockade of a number of cells are in agreement with our theoretical findings. The increase in the portion of bursting cells during HFS (Welter et al. 2004) was not observed in our single neuron model studied in this paper. Such phenomena might be due to mutual interactions within the network and, hence, beyond the scope of our simple single cell model.

In general, there are different stimulation methods and mechanisms that may cause a depolarization blockade, see e.g. Beurrier et al. (2001), Bowman and McNeal (1986), Bhadra and Kilgore (2005), Kilgore and Bhadra (2004), Kilgore and Bhadra (2006), Williamson and Andrews (2005), Woo and Campbell (1964). As analytically shown in Sect. 3, in our model, the HFS corresponding to the vibrational forces appears only in Eqs. (14b)–(14d) that govern the dynamics of the gating variables. Hence, in our model, HFS exclusively acts on the ion channels, so that—for sufficiently large stimulation frequency (compared to the neuron’s spontaneous frequency)—the membrane potential displays only high-frequency oscillations of moderate amplitude around a constant value close to the neuron’s resting potential. Put otherwise, HFS at sufficiently large stimulation frequencies induces a block of the voltage-gated ion channels. Accordingly, for sufficiently large stimulation frequency and stimulation amplitude, the oscillations of the STN model neuron’s membrane potential fall below the threshold amplitude $v_m$, so that the STN neuron will no longer be able to excite a postsynaptic GPe neuron (see Sect. 4). This is in accordance with what has been experimentally observed in rat STN slice (Beurrier et al. 2001), where short duration high-frequency stimulus trains effectively blocked the spontaneous neuronal STN activity due to a strong depression of intrinsic voltage-gated currents. In contrast, for smaller stimulation amplitudes, e.g., for a certain range of $A < A_{1:1}$ in Fig. 5b, the STN neuron performs oscillations that are still of larger amplitude and strongly influenced by the stimulation, so that any type of information processing will most probably be significantly hindered. This corresponds to the notion of the neuronal jamming (Benabid 2003; Benabid et al. 2005).

In Sect. 3, we revealed that our results are independent of the particular waveform. In the context of electrical stimulation of epileptiform activity in rat hippocampal slices, the relationship between stimulation effect and stimulus waveform was addressed by Bikson et al. (2001). In that study, sinusoidal high-frequency (2,050 Hz) electrical fields were delivered across rat hippocampal slices and turned out to block epileptiform activity, irrespective of how the epileptiform activity was induced (by zero calcium, low calcium, high potassium, or picrotoxin) (Bikson et al. 2001). Sinusoidal and square stimulus waveforms gave rise to the same (and statistically nondistinguishable) results (Bikson et al. 2001).

To study the impact of STN HFS on individual STN neurons, Meissner et al. (2005) performed single-unit recordings with four individually driven micro-electrodes in the STN network of MPTP-lesioned nonhuman primates during STN–HFS. They showed that STN–HFS leads to a decrease in the mean neuronal firing rate, but does not completely inhibit the firing of individual neurons. Rather only a small portion of the neurons was completely blocked. Another portion was not blocked; in fact, their mean firing rate was not modified by STN–HFS. The largest portion of STN neurons was physically blocked in the sense that their firing was blocked within the first 3 ms after each single electrical pulse (of a HF pulse train), and the probability of firing re-increased till the occurrence of the subsequent electrical pulse within the HF pulse train. The data by Meissner et al. (2005) do not show whether this differential type of blocking effect depends on the distance between stimulation electrode and neuron. In fact, our theoretical results do not contradict to the experimental results by Meissner et al. (2005): For instance, the portion of neurons that were completely blocked by the stimulation might be located close enough to the stimulation electrode. In that case, the STN–HFS might primarily exert an effect on the neuronal membranes, in accordance with our theoretical results.

In addition, there is also theoretical evidence supporting our results. Modolo et al. (2008) have numerically analyzed the impact of high-frequency DBS in two models, a simple firing rate model, initially developed by Gillies and Willshaw (2004) and extended by taking into account the impact of HFS (Modolo et al. 2008), and a population-based model. Both models refer to the “pacemaker-like” complex comprising STN and Globus Pallidus (GPe) (Plenz and Kital 1999). In the firing rate model, the amplitude of the limit cycle (corresponding to oscillatory firing) decreased as the stimulation amplitude of HFS increased (Modolo et al. 2008). In the population-based model, the bursting activity disappeared for sufficiently large stimulation frequency ($>$100 Hz) and amplitude (Modolo et al. 2008). HFS-induced suppression of neural and oscillatory dynamics has also been numerically observed in another network model of the STN and GPe (Hauptmann and Tass 2007) and in networks of phase oscillators (Lysyansky et al. 2011; Tass 2001).

A theoretical study (Rubin and Terman 2004) was devoted to the effects of monophasic HFS^1^ on a more complex neural network including the STN. In that study, HFS eliminates spontaneous low-frequency oscillations and forces STN neurons to trigger a spike at each HFS spike. In other words, spontaneous low-frequency oscillations are replaced by tonic, HFS-locked firing. Accordingly, Rubin and Terman (2004) interpreted their results as an indication of an HFS-induced increase in the firing rates of target cells, rather than an inhibitory effect. In contrast, in our model, the HFS-induced spiking of large amplitude is just an intermediate dynamical range observed provided the stimulation amplitude is not strong enough or the stimulation frequency is not high enough. In fact, for sufficiently large values of the stimulation frequency and amplitude in our study, we additionally observe the regime of nonspiking subthreshold oscillations, i.e., a dynamical regime, where the STN firing is suppressed (see Sect. 4).

Our analysis is based on simple neuron models that take into account only the properties of the membrane conductance, while the neuronal morphology is neglected. Nevertheless, the membrane conductance is the main ingredient responsible for the nonlinearity, which conditions the effect of the stabilization of the neuron resting state. The analysis of more complex multi-compartment neuron models under HFS will be the subject of future research. We plan to extend the averaging approach for such systems and simplify its numerical analysis by using averaged equations. Moreover, we believe that the averaged equations derived here for single-compartment neuron models provide a solid foundation for further studies of HFS effects in large-scale neural networks, since the high-frequency terms are eliminated. Such an extension of our approach may turn out to be fruitful, since the suppression of somatic firing within a target nucleus is not the only effect of HFS [for review see McIntyre et al. (2004a)]. In particular, in parallel to the blockade or inhibition of somatic firing, HFS may cause an independent activation of efferent axons of projection neurons (de-coupling of somatic and axonal activity) (Hashimoto et al. 2003; McIntyre et al. 2004a, b; Montgomery 2006). We do not claim that the somatic response dominates the overall network response to HFS. In fact, based on our approach and our findings, we cannot come up with such a claim. In contrast, our approach is different. In a first step, in this paper, we focus on the somatic response of a simple single STN neuron and derive its frequency-dependent characteristics. Intriguingly, some of its features resemble the characteristics of relevant clinical effects observed in patients. In a next step, we plan to increase the complexity of our model. This is to finally assess where characteristic response properties to HFS stem from and, hence, elucidate the differential dynamical contributions of different structures. Eventually, in a large-scale network, the somatic response of STN neurons might be of subordinate role for the generation of HFS responses, and the characteristics of the somatic STN responses observed in our paper might fit to clinical observations, but nevertheless might have to be considered as epiphenomenon. Still our approach might contribute to carve this out, in particular, also by applying the same type of approach employing averaged equations to models of different and increasing complexity. More complex and realistic network models might also contribute to assess to which degree STN neurons are actually modulated by electrical fields as opposed to other neuronal structures.

In our analysis, we used the method of averaging (Sanders et al. 2007) to derive an approximate analytical solution for the problem under study. In particular, we did not only use direct numerical simulations, because our analytical approach enabled us to reveal general statements concerning the underlying dynamical Kapitsa-type mechanism of action of HFS as well as the independence of this finding with respect to variations in the particular waveform of HFS. Our analytical approach enabled us to establish a universal conclusion about an action of HFS on a general HH-type neuron model. We have shown that for frequencies considerably larger than the reciprocal of the neuron’s characteristic time scale, the result of this action only depends on the ratio between the amplitude and the frequency of the stimulating signal.

## Footnotes

1 In contrast, we use biphasic charge-balanced stimulation, which is clinically mandatory to avoid tissue damage (Harnack et al. 2004; Piallat et al. 2009).
